# Atypical fracture of the ulna associated with alendronate use

**DOI:** 10.3109/17453674.2011.636676

**Published:** 2011-11-25

**Authors:** Kristian Bjørgul, Astor Reigstad

**Affiliations:** Department of Orthopaedic Surgery, Ostfold Hospital Trust, Fredrikstad, Norway

We report a case of a stress fracture of the ulna following 4 femoral fractures associated with bisphosphonate use. The patient (a woman born in 1928) had been taking bisphosphonates since 1994 when osteoporosis was diagnosed after a trochanteric fracture of the left femur. The fracture was treated with a sliding hip screw device (HCS) and healed uneventfully (Figure 1A). In 2001, she reported pain in her left thigh, and MRI images (now unavailable) reportedly showed a hairline fracture between screws 2 and 3 of the HCS construct. This hairline fracture was not visible on radiographs in 2003, which were taken due to persistent pain. In 2005, a radiograph revealed an osteopenic lesion of uncertain nature between screws 2 and 3 (Figure 1B), which prompted removal of the plate. 7 days after plate removal, a transverse fracture occurred at a site thought to correspond to the osteopenic area (Figure 1C). This was treated with a 6-hole HCS (Figure 1D). 5 months later, a new fracture occurred at the most distal screw (Figure 2A). The fracture was treated with an intramedullary nail (Figure 2B). Finally, she had a transverse fracture of the femoral neck at the site of the proximal locking screw, which was treated with a long-stemmed bipolar hip arthroplasty in 2006 (Figure 2D). All fractures eventually healed, but the patient continued to use crutches because of residual symptoms in the injured leg.

**Figure 1.**
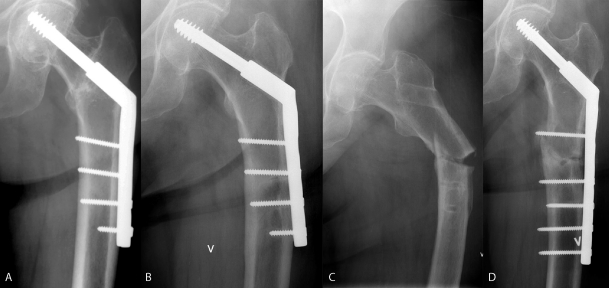
HCS from 1994 (panel A), initial painful lesion (B), post-removal fracture (C), and HCS for treatment of fracture (D).

**Figure 2.**
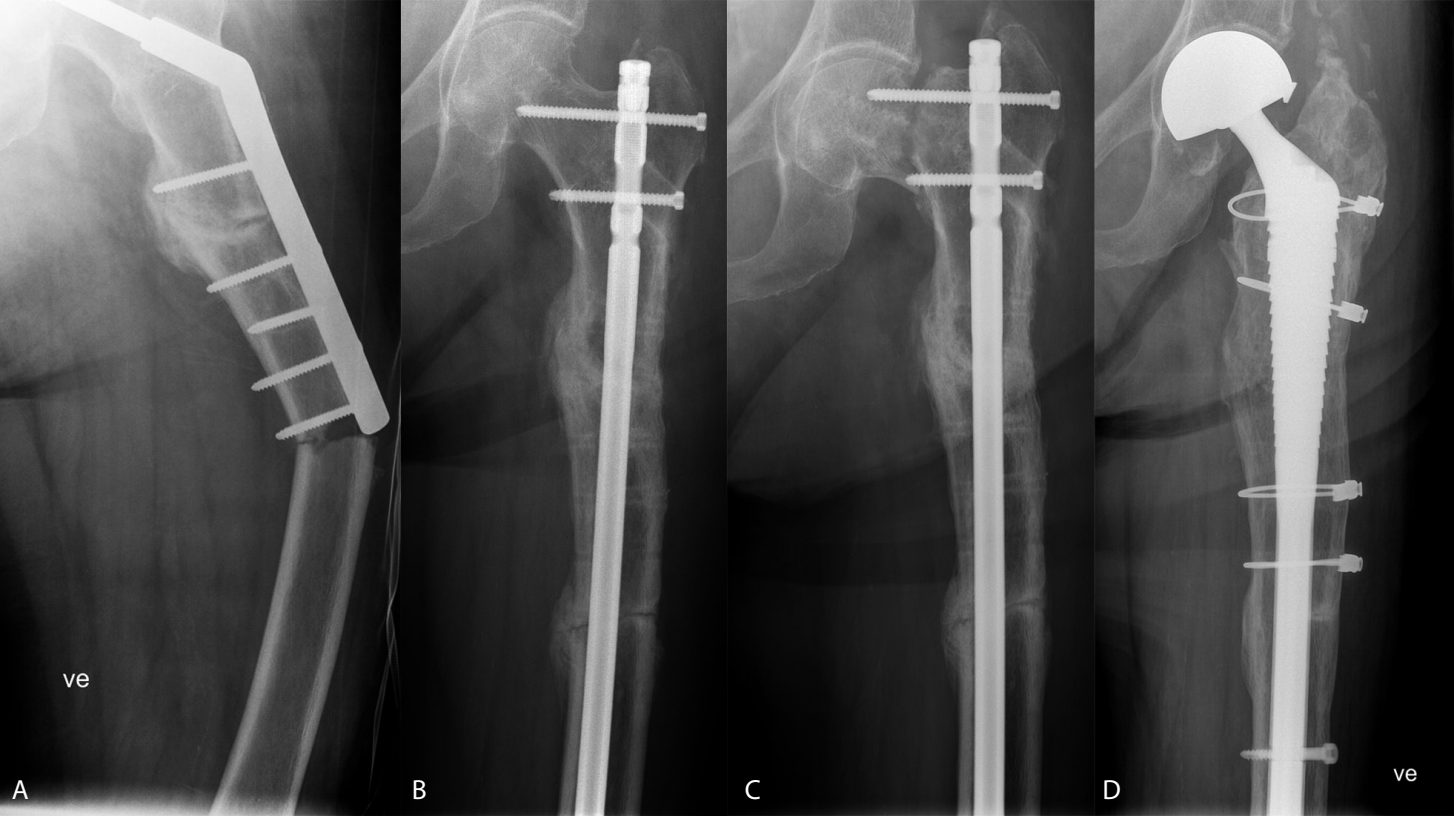
Implant-related fracture (panel A), treatment with intramedullary nail (B), failure of the neck of the femur and possible nonunion of diaphyseal fracture (C), and assumed definite treatment with long hydroxyapatite-coated stem (D).

In 2008, the patient noted pain in her left forearm, and radiographs showed sclerosis and a slight radiolucent line in the ulna proximal to a plated fracture sustained in 1991. After referral to our hospital, a stress fracture was diagnosed in 2010. We excised the fracture and fixed the ulna with a plate, and added bone graft from the ipsilateral radius (Figure 3). The fracture healed uneventfully.

**Figure 3.**
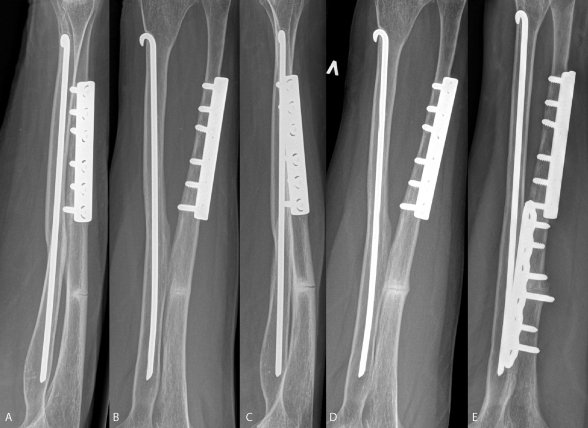
Front and lateral view from 2008 (panels A and B) and 2010 (C and D), and also postoperative view (E).

## Discussion

Stress fractures of the upper extremities are rare, but may occur in athletes (Sujino et al. 1998, Hamilton et al. 1999, Prisk and Hamilton 2008). Outside of the athletic population, stress fractures of the ulna have been associated with the use of crutches (Sorensen 1992, Amin et al. 2004). Recent evidence suggests that long-term use of alendronate may be associated with stress fractures (Capeci and Tejwani 2009, Black et al. 2010). Initially, reports documented fractures of the femur sustained with no or minor trauma, but recent reports have suggested that stress fractures may occur in the tibia (Breglia and Carter 2010) as well as the pelvis (Imai et al. 2007).

It is believed that long-term use of alendronate may cause altered bone metabolism, in which the bone becomes brittle. However, the exact mechanism that causes this to occur is not fully understood (Aspenberg et al. 2010). It is believed that the incidence of stress fractures is low (Schilcher and Aspenberg 2009, Black et al. 2010), and does not outweigh the benefits in the treatment of osteoporosis and subsequent reduction in the risk of hip fracture (Aspenberg 2008).

In addition to the ulnar stress fracture, this patient was treated for no less than 3 implant-related fractures of the left femur. The first implant-related fracture occurred at an unusual location in a stable implant-bone construct. However, the subsequent fractures may be explained by increased stress at the end of the HCS as well as an unfortunate placement of proximal locking screws in the intramedullary nail, which created weakness at the base of the femoral neck. Nevertheless, the fracture pattern is unusual and leads one to suspect that the femoral fractures may have been related to the use of alendronate as well.

It is crucial to thoroughly investigate any patient on alendronate treatment who complains of extremity pain, with the possibility of a stress fracture in mind.
